# Supplementary material for: Distinct Characteristics and Clinical Outcomes to Predict the Emergence of MET Amplification in Patients with Non-Small Cell Lung Cancer Who Developed Resistance after Treatment with Epidermal Growth Factor Receptor Tyrosine Kinase Inhibitors
Source: Cancers (Basel). 2021 Jun 21;13(12):3096. doi: 10.3390/cancers13123096 (PMC8234556; doi:10.3390/cancers13123096)
Supplement: Supplementary file 1 [file cancers-13-03096-s001.zip › cancers-1243609_supplementary/Table S1, Table S2.pdf]

Table S1. Patient characteristics based on MET status (first/second generation cohort, N = 124)

	**MET –** (N = 109) N (%)	**MET +** (N = 15) N (%)	*P* value
**Median age, years (range)**	60 (28–84)	62 (43–73)	0.549
**Sex**			
Male	41 (37.6%)	5 (33.3%)	
Female	68 (62.4%)	10 (66.7%)	0.748
**Smoking status**			
Current smoker	5 (4.6%)	0 (0.0%)	
Ex-smoker	29 (26.6%)	6 (40.0%)	
Never smoker	75 (68.8%)	9 (60.0%)	
**Median pack-year of smoking (interquartile range)**	0.0 (0.0 – 15.0)	0.0 (0.0 – 18.0)	0.663
**Previous TKI line**			
First line	93 (85.3%)	11 (73.2%)	
Second line	15 (13.8%)	3 (20.0%)	
Third line	1 (0.9%)	1 (6.7%)	0.194
**Founder *EGFR* mutation**			
Exon 19 deletion	60 (55.0%)	9 (60.0%)	
L858R	40 (36.7%)	5 (33.3%)	
Other mutations**	9 (8.3%)	1 (6.7%)	0.932
**Liver metastasis**			
No metastases	93 (85.3%)	7 (46.7%)	
Baseline metastases without progression	0 (0.0%)	3 (20.0%)	
Baseline metastases with progression	3 (2.8%)	4 (26.7%)	
Progression with new lesion	13 (11.9%)	1 (6.7%)	0.071*
**Brain metastasis**			
No metastases	29 (26.6%)	8 (53.3%)	
Baseline metastases without progression	5 (4.6%)	4 (26.7%)	
Baseline metastases with progression	47 (43.1%)	2 (13.3%)	
Progression with new lesion	24 (22.0%)	1 (6.7%)	< 0.001*
Not evaluated	4 (3.7%)	0 (0.0%)	

MET, mesenchymal epithelial transition factor; TKI, tyrosine kinase inhibitor; EGFR, epidermal growth factor receptor; SD, standard deviation.

*Between progression versus no progression

**Other mutations include exon 20 insertion (p.A767_V769dup) (N = 1), G719X (N = 2), L861Q (N = 4), and S768I (N = 2) in MET (-) group and G179S/L861Q (N = 1) in MET (+) group.

Table S2. Patient characteristics based on MET status (third generation cohort, N = 62)

	MET – (N = 47) N (%)	MET + (N = 15) N (%)	*P* value
**Median age, years (range)**	61 (40–81)	55 (28–76)	0.067
**Sex**			
Male	16 (34.0%)	8 (53.3%)	
Female	31 (66.0%)	7 (46.7%)	0.182
**Smoking**			
Current smoker	3 (6.4%)	1 (6.7%)	
Ex-smoker	8 (17.0%)	9 (60.0%)	
Never smoker	36 (76.6%)	5 (33.3%)	
**Median pack-year of smoking (interquartile range)**	0.0 (0.0 – 0.0)	4.0 (0.0 – 33.0)	0.205
**Previous TKI line**			
First line	0 (0.0%)	0 (0.0%)	
Second line	43 (91.5%)	11 (73.3%)	
Third line	4 (8.5%)	4 (26.7%)	0.068
**Founder *EGFR* mutation**			
Exon 19 deletion	28 (59.6%)	10 (66.0%)	
L858R	19 (40.4%)	5 (33.3%)	0.623
Other mutations	0 (0.0%)	0 (0.0%)	
**Liver metastasis**			
No metastases	32 (68.1%)	11 (73.3%)	
Baseline metastases without progression	1 (2.1%)	0 (0.0%)	
Baseline metastases with progression	1 (2.1%)	3 (20%)	
Progression with new lesion	13 (27.7%)	1 (6.7%)	0.817*
**Brain metastasis**			
No metastases	15 (31.9%)	8 (53.3%)	
Baseline metastases without progression	2 (4.3%)	3 (20.0%)	
Baseline metastases with progression	12 (25.5%)	3 (20.0%)	
Progression with new lesion	13 (27.7%)	1 (6.7%)	0.029*
Not evaluated	5 (10.6%)	0 (0.0%)	

MET, mesenchymal epithelial transition factor; TKI, tyrosine kinase inhibitor; EGFR, epidermal growth factor receptor; SD, standard deviation.

*Between progression versus no progression
