# Supplementary figures and images for: Distinct Characteristics and Clinical Outcomes to Predict the Emergence of MET Amplification in Patients with Non-Small Cell Lung Cancer Who Developed Resistance after Treatment with Epidermal Growth Factor Receptor Tyrosine Kinase Inhibitors
Source: Cancers (Basel). 2021 Jun 21;13(12):3096. doi: 10.3390/cancers13123096 (PMC8234556; doi:10.3390/cancers13123096)

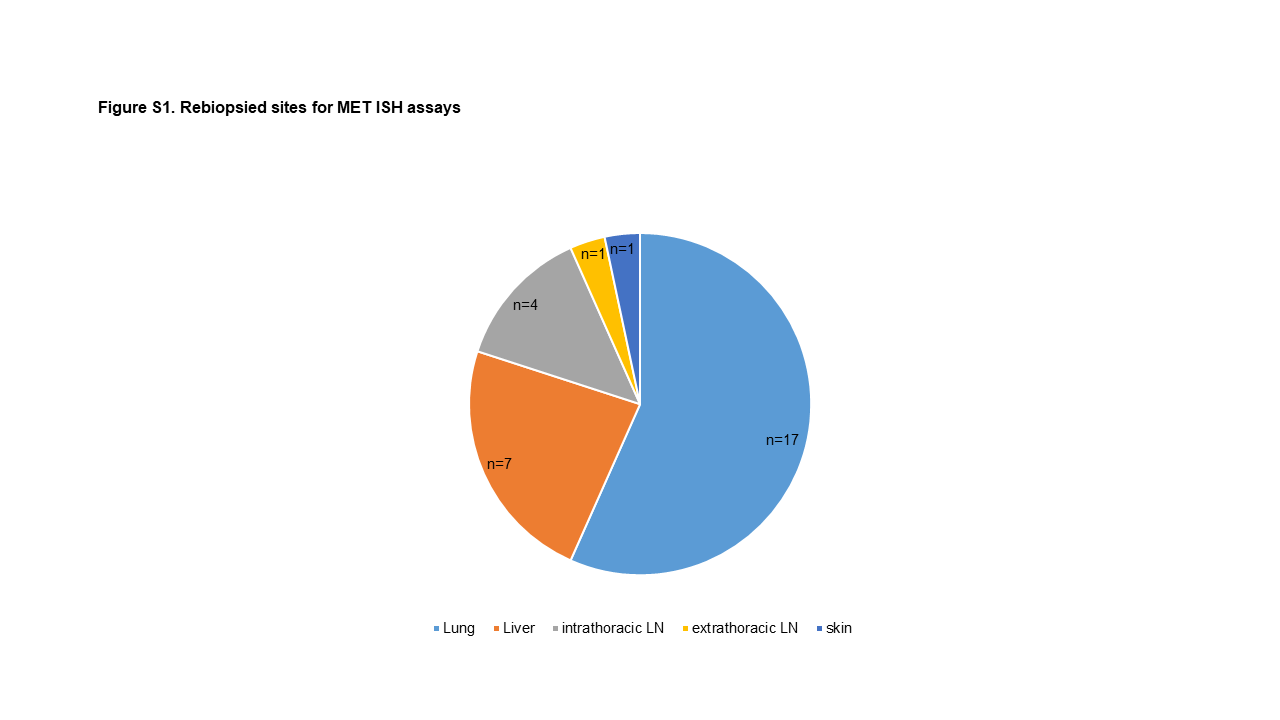

Supplement: Supplementary file 1 [file cancers-13-03096-s001.zip › cancers-1243609_supplementary/Figure S1.tif]
